# Expression and Clinical Significance of CXCR5 and LAG‐3 on Peripheral Blood CD8 ^+^ T Cells in Patients With Diffuse Large B‐Cell Lymphoma

**DOI:** 10.1002/kjm2.70005

**Published:** 2025-03-17

**Authors:** Xi‐Zhe Guo, Ya‐Fei Guo, Shi‐Xin Wu

**Affiliations:** ^1^ Department of Hematology The Second Affiliated Hospital of Fujian Medical University Quanzhou China

**Keywords:** CD8^+^ T cells, C‐X‐C chemokine receptor type 5, diffuse large B‐cell lymphoma, lymphocyte activation gene‐3, prognosis

## Abstract

Diffuse large B‐cell lymphoma (DLBCL) exhibits substantial biological and clinical heterogeneity. This study investigated the expression and prognostic implications of C‐X‐C chemokine receptor type 5 (CXCR5) and lymphocyte activation gene‐3 (LAG‐3) on peripheral blood CD8^+^ T cells in patients with DLBCL. A total of 71 DLBCL patients and 71 healthy controls were enrolled. The expression levels of CXCR5 and LAG‐3 on peripheral blood CD8^+^ T cells were assessed and analyzed for their impact on 5‐year progression‐free survival (PFS) and overall survival (OS). Results revealed significantly elevated CXCR5 and LAG‐3 expression levels in DLBCL patients compared to controls. CXCR5 expression correlated with lactate dehydrogenase (LDH) levels, extranodal involvement, Ann Arbor stage, and International Prognostic Index (IPI) scores, while LAG‐3 expression was associated with Eastern Cooperative Oncology Group (ECOG) scores, number of extranodal sites, bone marrow involvement, Ann Arbor stage, and IPI scores. Multivariate analysis identified advanced age, Ann Arbor stage III‐IV, and elevated CXCR5 and LAG‐3 expression as independent risk factors for poorer 5‐year PFS and OS. Furthermore, patients with higher CXCR5 and LAG‐3 expression levels demonstrated significantly reduced 5‐year PFS and OS rates. In conclusion, elevated CXCR5 and LAG‐3 expression on peripheral blood CD8^+^ T cells plays a pivotal role in DLBCL progression and prognosis, making these markers potential therapeutic targets or prognostic indicators.

## 1 Introduction

Diffuse large B‐cell lymphoma (DLBCL) is the most aggressive and heterogeneous subtype of non‐Hodgkin's lymphoma (NHL), accounting for ~40% of all NHL cases worldwide [1, 2]. Despite advances in treatment, including the standard first‐line rituximab plus CHOP (R‐CHOP) regimen and autologous stem cell transplantation (Auto‐SCT), the 5‐year progression‐free survival (PFS) and overall survival (OS) rates remain at 72.4% and 77.3%, respectively [3]. However, over 30% of DLBCL cases eventually progress to relapsed or refractory DLBCL [4] even with consolidation therapy, with a 5‐year relapse rate reaching 23.5% [5, 6, 7].

The International Prognostic Index (IPI), which stratifies patients based on serum lactate dehydrogenase (LDH) levels, age, performance status, disease stage, and the number of extranodal lesions, remains the most widely used prognostic tool for DLBCL. However, the IPI alone does not fully capture the underlying biological heterogeneity of DLBCL [8, 9]. This underscores the need for novel biomarkers that can provide a more comprehensive assessment of prognosis and guide the development of effective treatment strategies. Compared to tissue‐based markers, blood biomarkers offer advantages such as ease of sampling, non‐invasiveness, and high patient compliance, making them particularly valuable in identifying pre‐treatment indicators for clinical decision‐making in DLBCL.

Immune dysregulation is a critical driver of DLBCL pathogenesis and progression, with immune checkpoints playing a central role in suppressing T‐lymphocyte activity via receptor–ligand interactions [10, 11]. Immunophenotypic profiling has shown promise in predicting the prognosis of DLBCL [12, 13, 14, 15, 16]. Among immune checkpoints, lymphocyte activation gene‐3 (LAG‐3) emerges as a key immunosuppressive receptor that inhibits effector T‐cell function, promoting tumor immune escape [17]. Elevated LAG‐3 expression on CD8^+^ T lymphocytes has been observed in the tumor microenvironment of various solid tumors [18, 19]. In follicular lymphoma, LAG‐3 expression on tumor‐infiltrating lymphocytes under chronic immune stimulation has been linked to impaired CD8^+^ T lymphocytes [20].

Recent studies have highlighted the inhibitory effects of high LAG‐3 and PD‐1 expression on CD8^+^ T cells, reducing their tumor‐killing ability. Notably, combined blockade of LAG‐3 and PD‐1 has been shown to restore CD8^+^ T cell function, paving the way for personalized cellular immunotherapy for DLBCL [21]. These findings emphasize the need to explore LAG‐3 expression and its interaction with other markers, such as C‐X‐C chemokine receptor type 5 (CXCR5), to better understand their prognostic significance and therapeutic potential in DLBCL.

CXCR5, also known as CD185, is a seven‐transmembrane G protein‐coupled receptor protein [22]. While it is well‐established that CXCR5 is expressed on follicular helper T (Tfh) cells, B lymphocytes, and cytotoxic T lymphocytes, the role of CXCR5^+^ CD8^+^ T cells remains less explored compared to Tfh cells [23]. CXCR5^+^ CD8^+^ T cells are predominantly localized within the B‐cell zones and exhibit reduced expression of suppressive molecules, as demonstrated in murine models of chronic lymphocytic choriomeningitis virus infection [24]. The differentiation of CXCR5^+^ CD8^+^ T cells is regulated by the Bcl6‐Blimp1 axis, which plays a crucial role in controlling viruses within the B‐cell follicles, including Epstein–Barr virus (EBV)‐infected B cells and HIV‐infected Tfh cells [25]. These cells have also been identified as promising candidates for CD8^+^ T cell‐based immunotherapies, with evidence suggesting their potential to mediate the eradication of autologous tumor cells in patients with DLBCL. However, their function can be inhibited by immunosuppressive cytokines, such as IL‐10 [26]. CD8^+^ T lymphocytes are a pivotal component of the tumor microenvironment, serving as key effectors in tumor suppression and elimination. Despite their critical role, studies focusing on the expression and functional implications of CXCR5 and LAG‐3 on peripheral blood CD8^+^ T cells in DLBCL patients remain limited. This study aims to address this gap by investigating the expression patterns of CXCR5 and LAG‐3 on CD8^+^ T cells and exploring their clinical significance in the prognosis and treatment of DLBCL.

## 2 Materials and Methods

### 2.1 Ethics Statement

The study protocol was approved by the academic ethics committee of our hospital, and all procedures were conducted in accordance with the principles of the *Declaration of Helsinki*.

### 2.2 Study Subjects

Between February 2014 and January 2019, 96 DLBCL patients admitted to our hospital were retrospectively reviewed. Of these, 71 patients met the inclusion and exclusion criteria and were enrolled in the DLBCL group. An additional 71 healthy individuals undergoing physical examinations during the same period were included as the control group. There were no significant differences in body mass index (BMI), sex, or age between the two groups. The patient enrollment and sample analysis flow are shown in Figure S1.

### 2.3 Inclusion and Exclusion Criteria

Inclusion criteria were as follows: (1) Diagnosed with DLBCL according to the 2016 revision of the World Health Organization classification of lymphoid neoplasms [1], confirmed via morphological and immunohistochemical analysis conducted in the Department of Pathology at our hospital; (2) Complete medical records and clinical data available; (3) First‐time onset of the disease, with no prior radiotherapy or immunotherapy; and (4) Underwent regular R‐CHOP regimen treatment for 6–8 cycles in our hospital, without subsequent hematopoietic stem cell transplantation.

Exclusion criteria were as follows: (1) Complications with infectious disorders, other malignant tumors, or immunodeficiency disorders; (2) Severe organ dysfunction, including uremia, respiratory failure, hepatic failure, or cardiac failure; (3) Diagnosed with high‐grade B‐cell lymphoma, primary mediastinal large B‐cell lymphoma, or specific subtypes of DLBCL (primary central nervous system or reproductive system DLBCL), as well as DLBCLs transformed from indolent lymphoma, such as follicular lymphoma; and (4) Pregnant or lactating women.

### 2.4 Data Collection

Clinical data were retrieved from patient medical records, including information on age, BMI, sex, bone marrow involvement, Eastern Cooperative Oncology Group (ECOG) score, presence of B symptoms (e.g., pruritus, unexplained fever > 38°C, unintentional weight loss > 10% within 6 months, night sweats), extranodal involvement, spleen involvement, LDH levels, Ann Arbor stage, IPI score, and Hans classification. The IPI considers five factors: age, LDH level, ECOG score, extranodal sites, and Ann Arbor stage [8].

### 2.5 Peripheral Blood Sample Collection and Processing

Fasting venous blood (3 mL) was collected from DLBCL patients prior to treatment to isolate peripheral blood single nucleated cells (PBMCs) following standard protocols [27]: Blood samples were centrifuged at 2000 rpm/min for 10 min, with the supernatant aspirated in Eppendorf (EP) tubes. The remaining blood was mixed with an equal volume of phosphate buffer saline (PBS) (Biolegend, San Diego, CA, USA), gently layered onto lymphocyte separation medium (Becton Dickinson, Franklin Lakes, NJ, USA), and centrifuged at 2000 rpm/min for 20 min. After centrifugation, the layers formed were as follows (from top to bottom): plasma, milky white lymphocyte layer, clear separator layer, and erythrocyte layer. The lymphocyte layer was carefully extracted and transferred into a sterilized tube. The lymphocytes were washed twice with 4 mL PBS and centrifuged at 1000 rpm/min for 5 min. After two rinses, PBMCs were isolated. PBMCs were cryopreserved at—80°C for long‐term storage using the standard method (Figure S1). The collection of PBMCs was conducted with informed consent obtained from each participant.

### 2.6 Treatment and Follow‐Up

All patients newly diagnosed with DLBCL had not received any prior treatment before hospital admission. After admission, chemotherapy was initiated using the R‐CHOP regimen, which included rituximab (Day 1), cyclophosphamide (Day 2), adriamycin (Day 2), vincristine (Day 2), prednisone, or dexamethasone (Days 2–6). Chemotherapy cycles were repeated every 21 days, with patients receiving a total of 6–8 cycles. Treatment efficacy was evaluated based on the guideline criteria for NHL published by the National Comprehensive Cancer Network in 2015 [28].

Five‐year PFS and OS data were collected. PFS was defined as the time from diagnosis to either the end of follow‐up, disease recurrence, or death. OS was defined as the time from diagnosis to either the end of follow‐up or death due to any cause.

### 2.7 CXCR5 And LAG‐3 Detection

Cryopreserved PBMCs collected before treatment and during cycles 1–4 (weeks 3–12) were thawed. T lymphocytes were isolated using CD3‐APC staining. The isolated lymphocytes were divided into two groups for further analysis: Group 1: Stained with CD8‐FITC and CXCR5‐PE antibodies (20 μL/each). Group 2: Stained with CD8‐FITC and LAG‐3‐PE antibodies (20 μL/each). The samples were incubated at room temperature in the dark for 15 min and then washed twice with PBS. After resuspension, flow cytometry was performed using a FACSCalibur flow cytometer (Becton Dickinson) (Figure S1). Ratios of CD8^+^ CXCR5^+^ cells and CD8^+^ LAG‐3^+^ cells to CD8^+^ T cells were analyzed using CellQuest software (Becton Dickinson). All antibodies, including CD3‐APC, CXCR5‐PE, CD8‐FITC, and LAG‐3‐PE, were obtained from Biolegend.

### 2.8 Statistical Analysis

Data analyses and visualization were conducted using SPSS 27.0 statistical software (SPSS Inc, Chicago, IL, USA) and GraphPad Prism 9.5 software (GraphPad Software, San Diego, CA, USA). The Kolmogorov–Smirnov test was used to assess normality. Normally distributed measurement data were expressed as mean ± standard deviation, and inter‐group comparisons were performed using the independent sample *t*‐test. Non‐normally distributed data were represented as medians with quartiles (median [minimum, maximum]), with the Mann–Whitney U test employed for inter‐group comparisons. Categorical data were presented as numbers and percentages, with the Chi‐square test used for inter‐group analysis. A multivariate Cox regression model was constructed to evaluate the effects of CD8^+^ T cell CXCR5 and LAG‐3 expression on post‐treatment 5‐year PFS and OS in DLBCL patients. The Kaplan–Meier method was utilized for survival analysis, followed by the log‐rank test. Statistical significance was determined using a two‐sided test with a threshold of *p* < 0.05.

## 3 Results

### 3.1 Comparisons of Baseline Data

Based on the inclusion and exclusion criteria, 71 eligible patients were enrolled in the DLBCL group, and 71 healthy individuals were included as the Control group. There were no significant differences between the two groups regarding sex, BMI, or age (all *p* > 0.05), as summarized in Table 1.

**TABLE 1:** Clinical baseline characteristics.

Items		Control group (*n* = 71)	DLBCL group (*n* = 71)	*p*
Sex (male/female)		43/28	41/30	0.831
Age (years)		58.26 ± 8.16	57.30 ± 8.02	0.481
BMI (kg/m^2^)		22.55 ± 2.18	23.07 ± 2.71	0.210
Pathological subtype (cases)	GCB subtype	—	37	—
	non‐GCB subtype	—	34	—
LDH > upper limit of normal (cases)	No	—	32	—
	Yes	—	39	—
ECOG score (cases)	0 ~ 1	—	49	—
	2 ~ 4	—	22	—
B symptom (cases)	No	—	47	—
	Yes	—	24	—
Number of extranodal involvement > 1 (cases)	No	—	49	—
	Yes	—	22	—
Bone marrow involvement (cases)	No	—	66	—
	Yes	—	5	—
Ann Arbor stage (cases)	I‐II	—	26	—
	III‐IV	—	45	—
IPI score (cases)	0 ~ 2	—	42	—
	3 ~ 5	—	29	—

*Note*: BMI, body mass index; GCB, germinl center B‐cell‐like; LDH, lactate dehydrogenase; ECOG, Eastern Cooperative Oncology Group; IPI, International Lymphoma Prognostic Index. Count data were denoted as the number of cases and percentages, and inter‐group comparisons were performed by the Chi‐square test. Normally distributed measurement data were presented as the mean ± standard deviation, and inter‐group comparisons were enforced by independent sample *t*‐test.

### 3.2 CXCR5 And LAG‐3 Expression Was Augmented in Peripheral Blood CD8 ^+^ T Cells of DLBCL Patients

**FIGURE 1:**
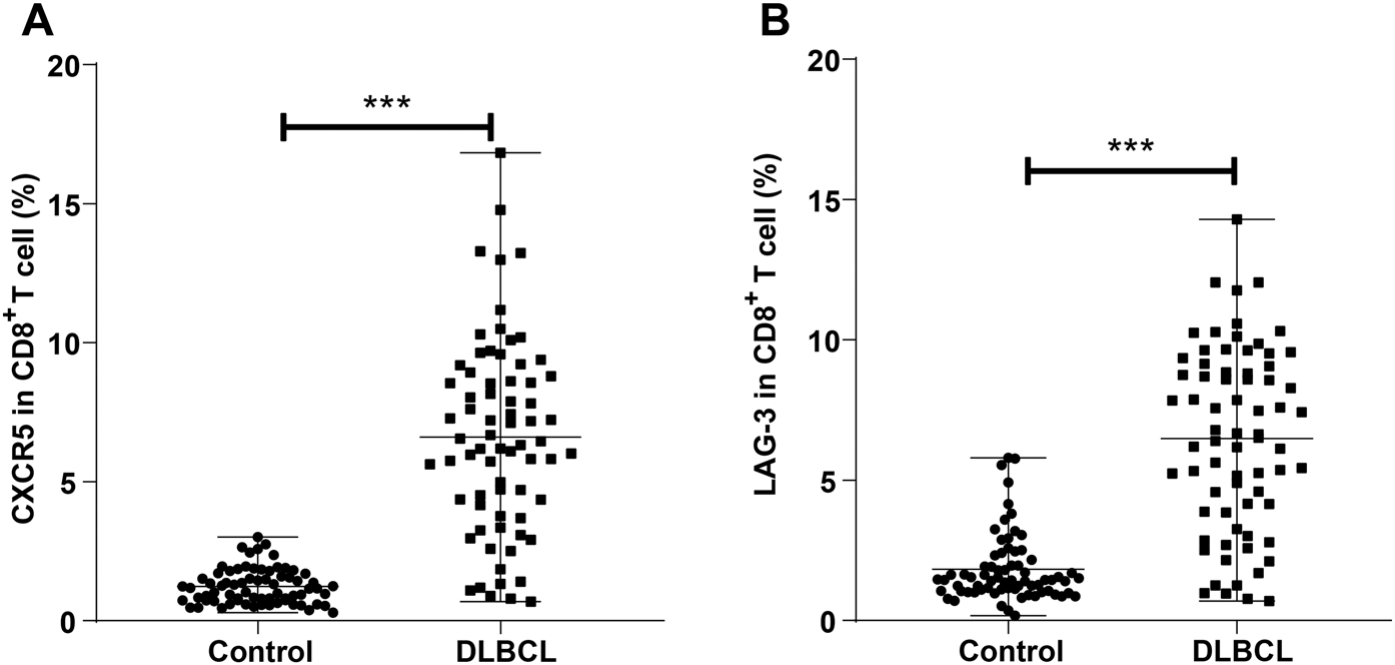
Expression rates of CXCR5 and LAG‐3 on peripheral blood CD8^+^ T cells in the two groups. A: Expression rate of CXCR5 on peripheral blood CD8^+^ T cells in the two groups; B: Expression rate of LAG‐3 on peripheral blood CD8^+^ T cells in the two groups. Data were described as quartile (median [minimum, maximum]), with inter‐group comparisons carried out by Mann–Whitney U test. ***p < 0.001.

Flow cytometry analysis was employed to evaluate the expression of CXCR5 and LAG‐3 in CD8^+^ T cells (gating strategy shown in Figure S2). The results revealed significantly elevated CXCR5 and LAG‐3 expression in the DLBCL group compared to the Control group (both *p* < 0.001, Figure 1).

### 3.3 Relationship of CXCR5 and LAG‐3 Expression in Peripheral Blood CD8 ^+^ T Cells With the Clinicopathologic Features of DLBCL Patients

The relationship between CXCR5 and LAG‐3 expression in peripheral blood CD8^+^ T and the clinicopathological characteristics of DLBCL patients was further explored. Elevated CXCR5 expression in CD8^+^ T cells was observed in patients with higher LDH levels, Ann Arbor stages (III‐IV), extranodal involvement exceeding one site, and an IPI score of 3–5 (*p* < 0.05). Similarly, increased LAG‐3 expression was associated with an ECOG score 2–4, bone marrow involvement, extranodal involvement exceeding one site, Ann Arbor stage of III‐IV, and an IPI score of 3–5 (*p* < 0.05). However, no significant differences in CXCR5 or LAG‐3 expression were noted based on B symptoms, sex, age, or pathological subtype (all *p* > 0.05). These findings suggest associations between CXCR5 expression and variables such as Ann Arbor stage, LDH levels, IPI score, and extranodal involvement, as well as between LAG‐3 expression and ECOG score, bone marrow involvement, Ann Arbor stage, extranodal involvement, and IPI score in DLBCL patients (Table 2).

**TABLE 2:** The relationship between the expression of CXCR5 and LAG‐3 on peripheral blood CD8^+^ T cells and clinicopathological features.

Items		Cases	CXCR5 expression rate (*n* = 71)	*p*	LAG‐3 expression rate (*n* = 71)	*p*
Sex	Male	41	6.67 ± 3.65	0.871	6.09 ± 3.45	0.229
	Female	30	6.53 ± 3.28		7.03 ± 2.89	
Age	> 60	26	6.73 ± 2.96	0.822	7.33 ± 3.41	0.093
	≤ 60	45	6.54 ± 3.77		5.99 ± 3.07	
Pathological subtype	GCB subtype	37	7.05 ± 4.12	0.270	6.78 ± 3.45	0.423
	non‐GCB subtype	34	6.13 ± 2.58		6.16 ± 3.02	
LDH > upper limit of normal	No	32	5.33 ± 2.76	0.004	5.80 ± 3.48	0.106
	Yes	39	7.66 ± 3.68		7.05 ± 2.95	
ECOG score	0 ~ 1	49	6.39 ± 3.51	0.431	5.92 ± 3.12	0.027
	2 ~ 4	22	7.10 ± 3.44		7.74 ± 3.22	
B symptom	No	47	6.32 (0.69, 16.83)	0.343	6.30 ± 3.30	0.507
	Yes	24	7.83 (0.79, 13.29)		6.85 ± 3.15	
Number of extranodal involvement > 1	No	49	5.99 ± 3.42	0.023	5.98 ± 3.27	0.047
	Yes	22	8.00 ± 3.25		7.62 ± 2.0.94	
Bone marrow involvement	No	66	6.56 ± 3.29	0.645	6.23 ± 3.15	0.017
	Yes	5	7.31 ± 5.88		9.78 ± 2.71	
Ann Arbor stage	I‐II	26	4.04 ± 2.27	< 0.001	5.17 ± 3.34	0.009
	III‐IV	45	8.10 ± 3.18		7.24 ± 2.96	
IPI score	0 ~ 2	42	5.85 ± 3.35	0.026	5.82 ± 3.21	0.036
	3 ~ 5	29	7.71 ± 3.41		7.45 ± 3.09	

*Note*: Count data were represented by the number of cases and percentages, with the Chi‐square test utilized for inter‐group comparisons. Measurement data conforming to normal distribution were signified as the mean ± standard deviation, and inter‐group comparisons were performed using the independent sample *t*‐test. Non‐normally distributed measurement data were expressed as quartiles (median [minimum, maximum]), and for inter‐group comparisons, the Mann–Whitney U test was utilized.

Abbreviations: ECOG, Eastern Cooperative Oncology Group; IPI, International Lymphoma Prognostic Index; LDH, lactate dehydrogenase.

### 3.4 Impact of CD8 ^+^ T Cell CXCR5 and LAG‐3 Expression Levels on the Prognosis in DLBCL Patients

To evaluate the prognostic impact of CXCR5 and LAG‐3 expression in CD8^+^ T cells on DLBCL patients, the post‐treatment 5‐year OS, and PFS rates were analyzed. Complete follow‐up data were available for all patients, with no cases lost to follow‐up. Among the 71 DLBCL patients, 33 cases experienced disease progression, yielding a 5‐year PFS of 54.17%, and 25 died, while 46 survived, resulting in a 5‐year OS of 64.79%. Univariate Cox regression analysis was performed using post‐treatment 5‐year PFS and OS (0 = survival/no disease progression, 1 = death/disease progression) as dependent variables. Independent variables included clinicopathological characteristics and CXCR5 and LAG‐3 expression levels. Factors significantly influencing 5‐year PFS and OS included age, ECOG score, Ann Arbor stage, extranodal/bone marrow involvement, LDH level, IPI score, and CXCR5 and LAG‐3 expression (*p* < 0.05). Multivariate Cox regression analysis, incorporating variables with *p* < 0.05 from the univariate analysis, identified the following independent risk factors for reduced 5‐year PFS and OS: Ann Arbor stage III‐IV, advanced age, elevated CXCR5, and LAG‐3 expression in CD8^+^ T cells (all *p* < 0.05, Table 3).

**TABLE 3:** Univariate and multivariate COX regression analyses of PFS and OS in DLBCL patients.

	PFS						OS					
Items	Univariate analysis			Multivariate analysis			Univariate analysis			Multivariate analysis		
	*p*	HR value	95% CI	*p*	OR value	95% CI	*p*	HR value	95% CI	*p*	HR value	95% CI
Sex	0.844	0.933	0.470 ~ 1.854	—	—	—	0.37	1.454	0.642 ~ 3.290	—	—	—
Age	0.002	1.083	1.029 ~ 1.139	0.003	1.104	1.034 ~ 1.178	0.001	1.099	1.038 ~ 1.164	0.001	1.155	1.061 ~ 1.257
Pathological subtype	0.135	1.703	0.847 ~ 3.426	—	—	—	0.496	1.316	0.597 ~ 2.899	—	—	—
B symptom	0.394	1.355	0.674 ~ 2.725	—	—	—	0.78	1.123	0.496 ~ 2.543	—	—	—
Bone marrow involvement	0.001	5.312	1.959 ~ 14.106	0.791	1.175	0.357 ~ 3.872	0.017	3.714	1.265 ~ 10.900	0.692	0.78	0.227 ~ 2.677
LDH level (> upper limit of normal)	0.014	2.631	1.221 ~ 5.671	0.352	1.628	0.583 ~ 4.544	0.047	2.426	1.012 ~ 5.814	0.511	1.471	0.465 ~ 4.653
ECOG score (2 ~ 4 points)	0.001	3.333	1.671 ~ 6.648	0.187	2.209	0.709 ~ 5.806	0	4.448	1.988 ~ 9.950	0.138	2.442	0.751 ~ 7.940
Number of extranodal involvement > 1	0.002	3.044	1.526 ~ 6.070	0.696	0.818	0.300 ~ 2.236	0.006	3.021	1.372 ~ 6.651	0.156	0.44	0.142 ~ 1.368
Ann Arbor stage (stage III‐IV)	0	13.89	3.312 ~ 58.252	0.014	7.678	1.501 ~ 39.285	0.004	19.646	2.653 ~ 145.494	0.046	9.709	1.040 ~ 90.613
IPI score (3 ~ 5 points)	0	4.079	1.961 ~ 8.483	0.567	0.654	0.153 ~ 2.802	0	6.209	2.464 ~ 15.650	0.6	1.581	0.286 ~ 8.730
CXCR5	0	1.253	1.133 ~ 1.385	0.012	1.174	1.035 ~ 1.330	0.004	1.172	1.051 ~ 1.308	0.018	1.194	1.031 ~ 1.383
LAG‐3	0	1.266	1.127 ~ 1.423	0.001	1.243	1.008 ~ 1.420	0	1.272	1.103 ~ 1.468	0.002	1.318	1.105 ~ 1.572

Abbreviations: 95% CI, 95% confidence interval; ECOG, Eastern Cooperative Oncology Group; HR, hazard ratio; IPI, International Prognostic Index; LDH, lactate dehydrogenase; OS, overall survival; PFS, progression‐free survival.

### 3.5 Reduced 5‐Year PFS and OS Were Found in DLBCL Patients With High CXCR5 and LAG‐3 Expression in CD8 ^+^ T Cells

**FIGURE 2:**
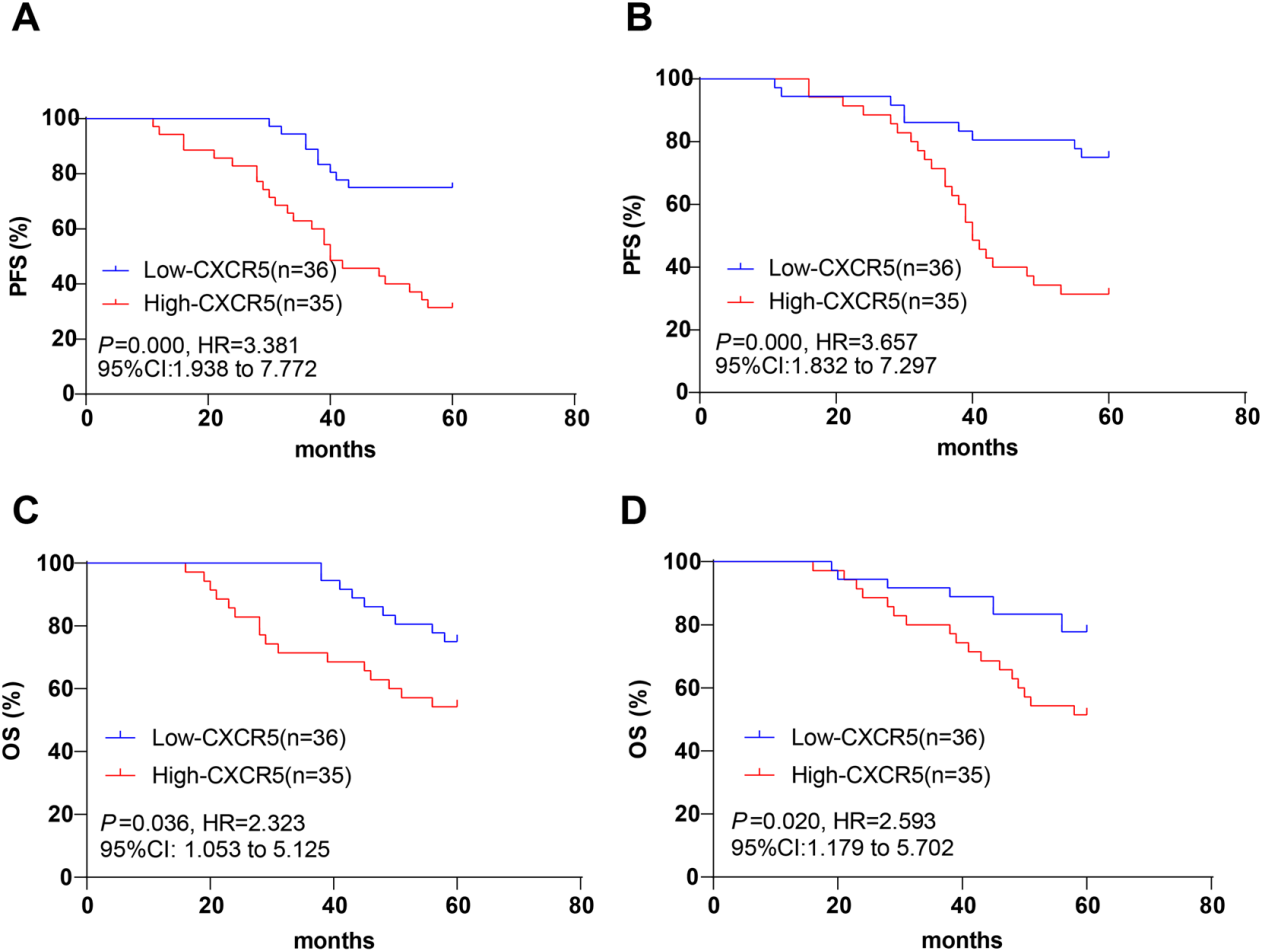
DLBCL patients with high CXCR5 and LAG‐3 expression in CD8^+^ T cells had decreased 5‐year PFS and OS. Kaplan–Meier method was exploited for analyzing the effects of CXCR5 and LAG‐3 expression in CD8^+^ T cells on 5‐year PFS and OS of DLBCL patients. PFS, progression‐free survival; OS, overall survival.

To further explore the impact of CXCR5 and LAG‐3 expression on patient outcomes, DLBCL patients were stratified into high‐CXCR5/low‐CXCR5 and high‐LAG‐3/low‐LAG‐3 groups based on median expression values: CXCR5^+^ CD8^+^ T (%) = 6.45% and LAG‐3+ CD8+ T (%) = 6.64%. Kapan–Meier curve analysis revealed that patients in the high‐CXCR5 and high‐LAG‐3 groups had significantly poorer outcomes compared to those in the low‐CXCR5 and low‐LAG‐3 groups, 5‐year PFS [significantly lower in high‐CXCR5 and high‐LAG‐3 groups] (*p* < 0.001, Figure 2A/B) and 5‐year OS [reduced in high‐CXCR5 and high‐LAG‐3 groups] (*p* < 0.05, Figure 2C/D).

## 4 Discussion

Patients with DLBCL who relapse after autologous stem cell transplantation (auto‐SCT) face poor prognoses, especially if relapse occurs within 1 year. Such patients have a 3‐year OS rate of only 20% [29], with no benefit from maintenance therapy using rituximab [30]. Recent evidence highlights the elevated expression of CXCR5^+^ CD8^+^ T cells and their cytotoxic role against autologous tumor cells in DLBCL [26]. This study provides novel insights into the expression and clinical significance of CXCR5 and LAG‐3 in peripheral blood CD8^+^ T cells of DLBCL patients, demonstrating that expression of these markers correlates with decreased 5‐year OS post‐treatment.

CXCR5 has been implicated in tumorigenesis and cancer progression [31]. Elevated CXCR5 expression is observed in primary central nervous system‐DLBCL and primary intraocular lymphoma [31]. LAG‐3, another critical target molecule, has emerged as a key target for immune checkpoint inhibitors in tumor immunotherapy [21]. Functionally, LAG‐3 suppresses T‐cell proliferation and activation, promotes immune exhaustion, and enhances the immunosuppressive effects of regulatory T cells (Tregs) [32]. It is well‐documented that upregulated LAG‐3 expression reduces cytokine production and T‐cell proliferation, resulting in T‐cell dysfunction during tumor stimulation and chronic immune challenges [33]. Furthermore, increased LAG‐3 and PD‐1 expression in DLBCL tissues and peripheral blood CD8^+^ T cells has been linked to impaired tumor cell killing [21]. Consistently, our study identified elevated CXCR5 and LAG‐3 expression on CD8^+^ T cells in DLBCL patients.

Our findings also revealed correlations between CXCR5 and LAG‐3 expression and specific clinicopathologic features of DLBCL. Elevated CXCR5 expression was associated with high LDH levels, advanced Ann Arbor stage (III‐IV), extranodal involvement (> 1 site), and higher IPI scores. Similarly, increased LAG‐3 expression correlated with elevated ECOG scores (2–4), advanced Ann Arbor stage, bone marrow involvement, extranodal involvement (> 1 site), and IPI scores. Interestingly, neither CXCR5 nor LAG‐3 expression showed a significant relationship with age‐related factors within the IPI score.

The IPI score, which assesses prognosis based on five factors: patient age (> 60), serum LDH level (> upper normal limit), ECOG score (2–4), Ann Arbor stage (III‐IV), and extranodal involvement (> 1 site), is widely used for risk stratification in DLBCL [8, 34, 35]. Our findings suggest that assessing CXCR5 and LAG‐3 expression in peripheral blood CD8^+^ T cells in peripheral blood CD8^+^ T cells could provide additional prognostic value beyond the traditional IPI score, potentially offering a novel approach for risk stratification in DLBCL patients.

Notably, LAG‐3 expression in tumor‐infiltrating lymphocytes has been correlated with LDH levels and bone marrow involvement in prior studies [20]. Similarly, CXCR5^+^FoxP3^+^ follicular regulatory T cells have been positively associated with LDH levels in breast cancer (BC) [36]. These reports align with our findings, reinforcing the significance of CXCR5 and LAG‐3 expression in relation to specific clinicopathologic features of DLBCL. Collectively, these data underscore the potential of CXCR5 and LAG‐3 as biomarkers for prognosis and therapeutic targets in DLBCL.

Elevated LAG‐3 expression has been linked to poor prognosis in follicular lymphoma [37]. Similarly, variables, such as CXCR3 and CXCR5, have been suggested as prognostic indicators in peripheral T‐cell lymphoma, not otherwise specified (PTCL‐NOS). Nevertheless, the IPI score and its variants remain the most reliable prognostic tools [38]. Additionally, Jiang et al. reported that high CXCR5 and CXCL13 expression levels are associated with decreased 5‐year OS in BC, suggesting their potential diagnostic and prognostic biomarkers [39]. In colorectal carcinoma, patients high percentage of LAG‐3^+^ cells exhibit shorter survival period [40].

Consistent with these findings, our univariate and multivariate COX regression analyses identified Ann Arbor stage (III‐IV), high CXCR5 and LAG‐3 expression rates on CD8^+^ T cells, and advanced age as independent risk factors for 5‐year PFS and OS in DLBCL patients. Kaplan–Meier analyses further confirmed that DLBCL patients with elevated CXCR5 and LAG‐3 expression exhibited significantly lower post‐treatment 5‐year PFS and OS compared to those with lower expression levels.

In summary, this study highlights the prognostic significance of elevated CXCR5 and LAG‐3 expression on peripheral blood CD8^+^ T cells in DLBCL patients. However, several limitations must be acknowledged. First, as a single‐center retrospective study with a relatively small sample size, our findings require validation through larger, multi‐center studies. Additionally, the lack of prospective external validation limits the generalizability of our conclusions. Future prospective studies are necessary to substantiate the clinical utility of CXCR5^+^ and LAG‐3^+^ CD8^+^ T‐cell immunotypes as prognostic biomarkers in DLBCL.

Another limitation lies in the scope of our investigation. This study focused solely on the expression of CXCR5 and LAG‐3 in peripheral blood CD8^+^ T cells, as we were unable to obtain lymphoma and normal lymphoid tissue samples. Consequently, the expression levels of these markers in tumor‐infiltrating lymphocytes remain unexplored. Further research should examine CXCR5 and LAG‐3 expression in lymphoid tissues to better understand their role in the tumor microenvironment.

Finally, while our findings suggest that CXCR5 and LAG‐3 may serve as prognostic biomarkers, their potential as therapeutic targets for DLBCL remains unclear. Future studies should investigate whether targeting these markers could improve clinical outcomes in DLBCL patients. Expanding the sample size and conducting multi‐center studies will be crucial to enhancing the robustness and credibility of our findings and exploring the therapeutic implications of CXCR5 and LAG‐3 in this context.

## Ethics Statement

The experiments were authorized by the academic ethics committee of the second affiliated Hospital of Fujian Medical University. All procedures adhered to the *Declaration of Helsinki*.

## Conflicts of Interest

The authors declare no conflicts of interest.

## Supporting information


**Figure S1.** The patient enrollment and sample analysis flow.

**Figure S2.** Gating strategy for detecting CXCR5/LAG‐3 expression in peripheral blood CD8^+^ T cells using flow cytometry.

## Data Availability

The data that support the findings of this study are available from the corresponding author upon reasonable request.
