# Supplementary figures and images for: Expression and Clinical Significance of CXCR5 and LAG‐3 on Peripheral Blood CD8 + T Cells in Patients With Diffuse Large B‐Cell Lymphoma
Source: Kaohsiung J Med Sci. 2025 Mar 17;41(5):e70005. doi: 10.1002/kjm2.70005 (PMC12087394; doi:10.1002/kjm2.70005)

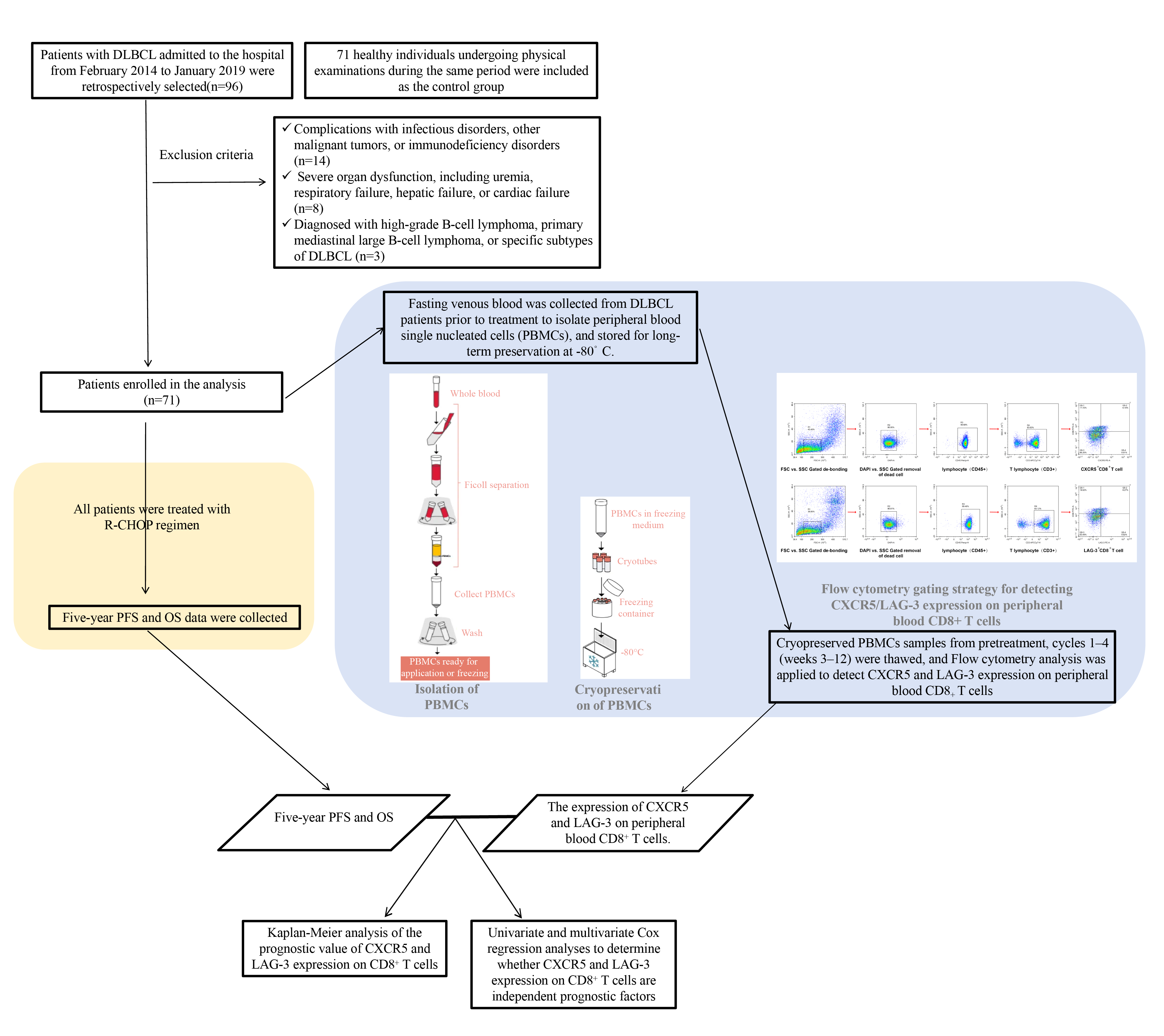

Supplement: Supplementary file 1 — Figure S1. The patient enrollment and sample analysis flow. [file KJM2-41-e70005-s002.tif]

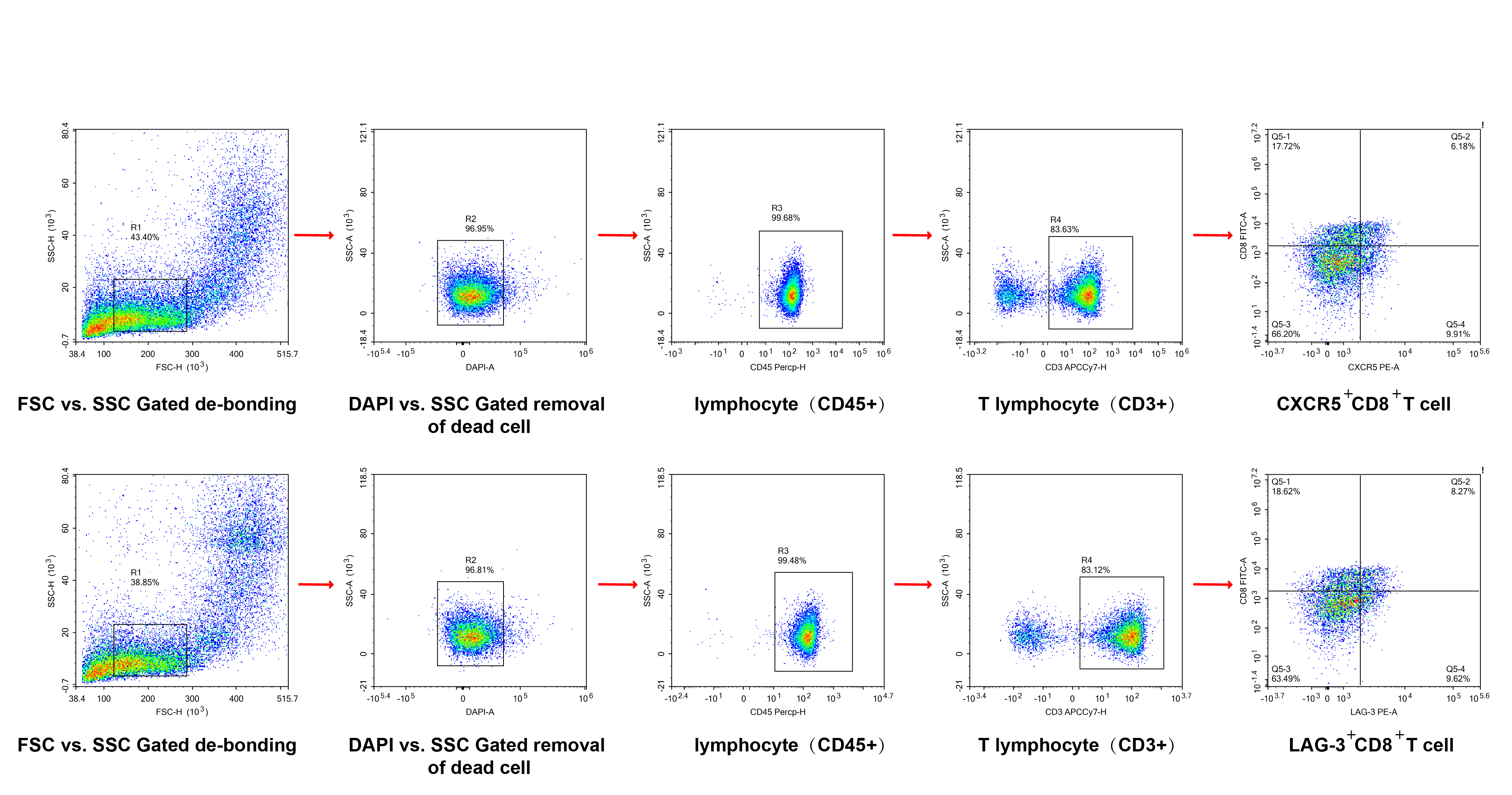

Supplement: Supplementary file 2 — Figure S2. Gating strategy for detecting CXCR5/LAG‐3 expression in peripheral blood CD8+ T cells using flow cytometry. [file KJM2-41-e70005-s001.tif]
